# *PNPT1* Spectrum Disorders: An Underrecognized and Complex Group of Neurometabolic Disorders

**DOI:** 10.3390/muscles3010002

**Published:** 2024-01-19

**Authors:** Paulo Sgobbi, Igor Braga Farias, Paulo de Lima Serrano, Bruno de Mattos Lombardi Badia, Hélvia Bertoldo de Oliveira, Alana Strucker Barbosa, Camila Alves Pereira, Vanessa de Freitas Moreira, Marco Antônio Troccoli Chieia, Adriel Rêgo Barbosa, Pedro Henrique Almeida Fraiman, Vinícius Lopes Braga, Roberta Ismael Lacerda Machado, Sophia Luiz Calegaretti, Isabela Danziato Fernandes, Roberta Correa Ribeiro, Marco Antonio Orsini Neves, Wladimir Bocca Vieira de Rezende Pinto, Acary Souza Bulle Oliveira

**Affiliations:** 1 Division of Neuromuscular Diseases, Neurometabolic Unit, Division of Neuromuscular Diseases, Federal University of São Paulo (UNIFESP), São Paulo 04039-060, SP, Brazil; paulo.sgobbi@unifesp.br (P.S.);; 2 Discipline of Clinical Semiology, Faculty of Medical Sciences of Nova Iguaçu, Nova Iguaçu 26260-045, RJ, Brazil

**Keywords:** inherited metabolic disorders, spinocerebellar ataxia, spastic ataxia, optic atrophy, mitochondrial disease, *PNPT1*

## Abstract

An 18-year-old man presented with slowly progressive infancy-onset spasticity of the lower limbs and cerebellar ataxia, associated with painless strabismus, intellectual disability, urinary incontinence, bilateral progressive visual loss, and cognitive decline since early adolescence. A neurological examination disclosed spastic dysarthria, left eye divergent strabismus, bilateral ophthalmoparesis, impaired smooth pursuit, severe spastic paraparesis of the lower limbs with global brisk tendon reflexes, bilateral extensor plantar responses, and bilateral ankle clonus reflex. Bilateral dysdiadochokinesia of the upper limbs, Stewart-Holmes rebound phenomenon, bilateral dysmetria, and a bilateral abnormal finger-to-nose test were observed. Markedly reduced bilateral visual acuity (right side 20/150, left side 20/400) and moderate to severe optic atrophy were detected. Neuroimaging studies showed cerebellar atrophy and bilateral optic nerves and optic tract atrophy as the main findings. As a complicated Hereditary Spastic Paraplegia, autosomal dominant Spinocerebellar Ataxia, or inherited neurometabolic disorders were suspected, a large next-generation sequencing-based gene panel testing disclosed the heterozygous pathogenic variant c.162-1G>A in intron 1 of the *PNPT1* gene. A diagnosis of *PNPT1*-related spastic ataxia was established. Clinicians must be aware of the possibility of *PNPT1* pathogenic variants in cases of spastic ataxia and spastic paraplegias that are associated with optic atrophy and marked cognitive decline, regardless of the established family history of neurological compromise.

## 1. Introduction

Hereditary ataxias and Hereditary Spastic Paraplegias (HSPs) represent significant groups of inherited neurological disorders that are associated with different clinical presentations, genetic inheritance patterns, and systemic and neurological complications. Among early-onset hereditary ataxias, autosomal recessive ataxias related to various etiologies, including neurodegenerative, neurodevelopmental, hereditary metabolic, and mitochondrial causes, stand out. These conditions are typically associated with an autosomal recessive inheritance pattern and often involve systemic complications [1,2]. However, it is essential to also consider the possibility of early-onset presentations with an autosomal dominant family history, without genetic anticipation, and seemingly de novo cases, which may be related to Spinocerebellar Ataxias (SCAs), described mainly in the last two decades. These SCAs are not related to trinucleotide repeat expansion mechanisms but instead to specific missense or nonsense variants. These SCA presentations often involve systemic, otoneurologic, and particularly neuro-ophthalmic dysfunctions [3]. 

Early-onset HSP is related to both pure and complicated presentations, with complicated forms being more frequently associated with autosomal recessive inheritance patterns and the occurrence of several central and peripheral neurological manifestations, sensorineural deafness, and neuro-ophthalmic complications, such as eyelid ptosis, ophthalmoparesis, retinitis pigmentosa, and optic atrophy [4,5,6]. Additionally, there is the rare group of spastic ataxias (SPAXs), mostly of autosomal recessive inheritance, with clinical manifestations appearing early in infancy or childhood onset and an association with different neuroradiological, systemic, and neuro-ophthalmic findings [7,8]. In addition, it is important to consider inherited neurometabolic disorders and primary mitochondrial diseases, especially those related to pathogenic variants in the nuclear genome [9,10,11,12]. 

Herein, we present the case report of a Brazilian patient with early-onset spastic ataxia that was slowly progressing, associated with cognitive decline and optic atrophy, in the absence of a significant family history. A comprehensive diagnostic approach was applied sequentially, culminating in genetic testing with a broad genetic panel, allowing for the specific diagnosis of a rare mitochondrial presentation linked to the nuclear genome. 

## 2. Case Presentation

An 18-year-old man presented to our Unit with a slowly progressive history of gait unsteadiness and infancy-onset spasticity involving the lower limbs. His clinical picture also included painless strabismus of the left eye and moderate intellectual disability since childhood. Bilateral progressive visual loss and marked cognitive decline since early adolescence were also observed. During adolescence, he developed also urinary incontinence and intestinal constipation. He became wheelchair-bound during late adolescence, despite maintaining limited ambulation in the orthostatic posture. Family history was unremarkable. His brothers had no neurological complaints. Parental consanguinity was not present. Prenatal and neonatal history did not reveal significant clinical events, and there were no relevant clinical or surgical complications.

Neurological examination disclosed mild spastic dysarthria, left eye exotropia (manifest divergent strabismus of the left eye), bilateral reduction in eye adduction during horizontal conjugate gaze, impaired smooth pursuit, severe spastic paraparesis of the lower limbs (modified Ashworth scale grade 4), global brisk tendon reflexes, bilateral extensor plantar responses (bilateral Babinski’s sign), bilateral Trömner and Hoffmann signs, bilateral ankle clonus reflex, mild bilateral dysdiadochokinesia of the upper limbs, positive bilateral Stewart-Holmes rebound phenomenon, bilateral dysmetria, and bilateral abnormal finger-to-nose test. No tongue atrophy or fasciculation was observed. No pseudobulbar signs were observed during clinical assessment. Ophthalmological examination showed markedly reduced bilateral visual acuity: right side 20/150, left side 20/400. Funduscopic evaluation showed moderate to severe optic nerve pallor (optic atrophy) and diffuse vascular attenuation in both eyes. Assessment of general aspects related to cerebellar ataxia and spasticity disclosed the following performance: (i) Scale for the Assessment and Rating of Ataxia (SARA) [13]: 27; (ii) International Cooperative Ataxia Rating Scale (ICARS) [14]: total ataxia score 71 (Oculomotor: 4; Dysarthria: 3; Kinetic: 39; Posture and Gait: 25); and (iii) Spastic Paraplegia Rating Scale (SPRS): 42 (Brazilian Portuguese validated version) [15]. 

**Figure 1:**
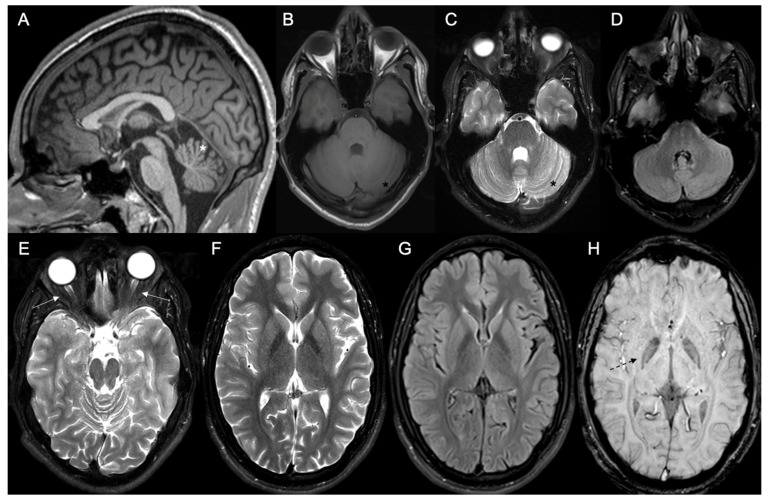
Brain MRI study of the proband at age 18 years old. (**A**) Sagittal brain MRI showing cerebellar atrophy (white asterisk) and normal corpus callosum in T1-weighted sequence. Axial brain MRI disclosing atrophy of the vermis and cerebellar hemispheres (black asterisks) in T1 (**B**), T2 (**C**), and FLAIR sequences (**D**). (**E**) Axial brain MRI showing mild atrophy of the optic nerves (white arrows) in T2-weighted imaging. No basal ganglia signal changes were observed in T2 (**F**) and FLAIR sequences (**G**). (**H**) Axial brain MRI showing hypointense sign in medial globus pallidus (black arrow) in SWI sequence.

**Figure 2:**
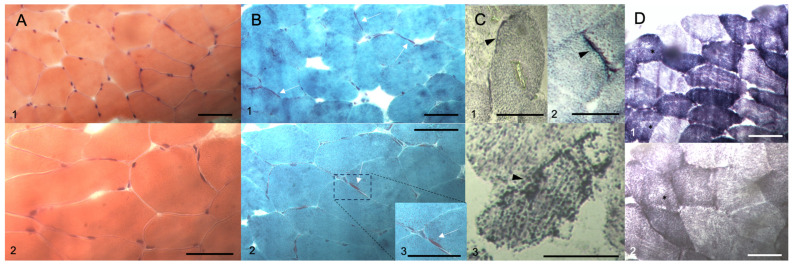
Deltoid muscle biopsy study. (**A**) Hematoxylin and Eosin-stained tissue section disclosed mild variation in muscle fiber caliber and no detectable abnormalities in the conjunctive tissue. (**B**) Modified Gömöri trichrome stain disclosed mild subsarcolemmal mitochondrial proliferation (white arrows; 1, 2, 3). (**C**) Succinate dehydrogenase (SDH) stain showed several foci of subsarcolemmal mitochondrial proliferation (black arrowheads; 1, 2, 3). (**D**) Nicotinamide adenine dinucleotide tetrazolium reductase (NADH-TR) staining disclosed areas of scattered moth-eaten appearance in muscle fibers (black asterisks; 1, 2). Size scale bars (**A**–**D**): 50 μm.

Lab exams were almost unremarkable, except for mildly raised serum creatine kinase levels (240–360 Ui/L; reference values of normality: up to 180 Ui/L). A basic metabolic screening for Inborn Errors of Metabolism (IEM), which included a quantitative panel of acylcarnitine on dried-blood spot, quantitative analysis of urinary organic acids, and plasma amino acid chromatography, did not reveal any significant pathological abnormalities. Cerebrospinal fluid analysis was also normal. Brain MR imaging (MRI) studies disclosed mild to moderate cerebellar atrophy; mild atrophy of the optic chiasma, optic nerves, and optic tract; and mild hypointense sign bilaterally in the medial globus pallidus in Susceptibility Weighted Imaging (SWI) sequence (Figure 1). No significant white matter changes were detected. Normal spectroscopy pattern was observed in cortical and subcortical regions and basal ganglia. Nerve conduction studies and needle electromyography evaluation were unremarkable. Deltoid muscle biopsy disclosed mild neurogenic amyotrophy and mild subsarcolemmal mitochondrial proliferation (Figure 2). 

Next-generation sequencing (NGS)-based multigene panel testing (including 600 genes related to inherited retinal disorders, cerebellar ataxias, HSP, nuclear mitochondrial diseases, peroxisomal, and lysosomal storage diseases) was performed. Genomic DNA was obtained from the proband and enriched for the targeted regions with a hybridization protocol, and then sequenced with the Illumina technology. All targeted regions were sequenced with >50× depth, and reads were aligned to the corresponding GRCh37 reference sequence. Genomic changes and variants were, then, properly identified and interpreted. Genetic testing disclosed the heterozygous pathogenic variant c.162-1G>A in intron 1 of the *PNPT1* gene (reference transcript: NM_033109.5), located on 2p16.1. This variant potentially affects acceptor splice site, leading to an abnormal RNA splicing process and loss-of-function impact [16]. This variant fulfilled PVS1, PM2, and PP5 criteria, according to the American College of Medical Genetics and Genomics (ACMG) and the Association for Molecular Pathology (AMP) recommendations [17]. This variant has been recently included in the ClinVar database (RCV002024472.2). It is absent in the gnomAD v.2.1.1. database. This variant’s TraPv3 score (Transcript-inferred Pathogenicity Score) was 0.519 (above the 97.5th percentile). In silico prediction tools are indicative of deleterious effect, including BayesDel addAF (score: 0.625), BayesDel noAF (score: 0.66), dbscSNV (Ada score: 0.9999, RF score: 0.94), EIGEN (score: 1.0766), GenoCanyon (score: 1.000), FATHMM (score: 0.9811), MutationTaster (score: 1.000), and DANN (score: 0.9957). PhastCons100way (score 1.000) and PhyloP100way (score 7.001) disclosed moderate to high conservation scores (other mammalian, primate, and vertebrate rank scores showed similar profiles for conservation). Other gene variants of uncertain significance were observed, all in heterozygous state, in *ADAMTS18* (c.2408G>A), *CDH23* (c.5578A>G), *FH* (c.883G>A), *SLC24A1* (c.3160T>G), *BBS4* (c.1511C>T), and *TMEM70* (c.554A>G) genes, but none of them had a direct correlation with the patient’s phenotype, and there was not an unequivocal deleterious profile by the several “in silico” prediction tools. No other family members were evaluated by genetic testing during diagnostic work-up. A definite diagnosis of *PNPT1*-related spectrum disorder was established, presenting with features that are similar to SCA type 25 (SCA25). 

In the context of motor rehabilitation treatment, periodic quarterly or semi-annual applications of botulinum toxin were recommended and performed with a transient symptomatic improvement lasting approximately three months after each application. These approaches specifically targeted components that are related to spasticity, alleviating pain, reducing immobility, preventing muscle atrophy due to disuse, and enhancing personal hygiene [18]. Additionally, baclofen was used as an adjunct therapy, administered orally, with a total daily dose of up to 60 mg, achieving an average dosage of 30 mg per day without significant adverse events and providing a mild improvement in spasticity. As specific disease-modifying therapies are not available for this neurogenetic condition, our main therapeutic goal was to ensure better functionality and quality of life and to adjust symptomatic treatments to the patient. Physical therapy (kinesiotherapy) was also prescribed, focusing on gait training, balance, and body posture, which played a crucial role in enhancing patient mobility and functionality. Regular clinical follow-up was also advised, with a focus on neuro-ophthalmology and otoneurologic clinics, along with an annual screening for cardiologic complications that are potentially associated with the underlying condition. Additionally, respiratory assessment was conducted regularly due to the possible neuromuscular restrictive ventilatory impairment. 

## 3. Discussion 

Pathogenic variants that are related to the *PNPT1* gene (2p16.1) have previously been correlated with central and peripheral neurological presentations with a variable age at onset in both sporadic and autosomal recessive and dominant familial contexts. *PNPT1* encodes polyribonucleotide nucleotidyltransferase 1, a phosphate-dependent 3′-to-5′ exoribonuclease enzyme or a polynucleotide phosphorylase (PNPase) involved in RNA degradation and processing by phosphorolysis, resulting in the generation of nucleotide diphosphates [19,20,21]. This protein is located mainly in the outer mitochondrial membrane and is also associated with the mitochondrial import of RNAs for further processing, particularly within the mitochondrial intermembrane space, and in the formation of complexes that are involved in the degradation of double-stranded RNA. Abnormal mitochondrial RNA (mtRNA) transport, trafficking, and processing lead to insufficient formation of mature mtRNA and secondary deficiency of oxidative phosphorylation components [19,20,21]. Abnormal function, resulting from loss-of-function variants, leads to structural alterations, trimerization, and hypofunction. These changes give rise to widespread central and peripheral dysfunction, notably affecting the sensory hair cells in the organ of Corti and the cochlear spiral ganglion neurons [22,23,24,25,26,27]. PNPase dysfunction has a strong and well-established correlation with the pathophysiology of interferonopathies, hearing loss, SCA, and primary and secondary oxidative phosphorylation disturbances [19,24,25,26,27]. 

The most significant autosomal dominant or sporadic form that is related to heterozygous pathogenic variants in the *PNPT1* gene is SCA25 (MIM #608703) [24]. SCA25 belongs to Group I in the classification of autosomal dominant cerebellar ataxias (ADCAs), proposed by Harding [28]. In most cases, it exhibits incomplete penetrance and variable intrafamilial and interfamilial expressivity. While an increasing number of cases are being recognized, the major families who were studied from clinical and genetic perspectives are predominantly from the Southern French and Australian regions. Some authors postulate the hypothesis that there is an abnormal accumulation of mitochondrial double-stranded RNA that leaks into the cytosol, leading to the hyperactivation and transcription of interferon-stimulated genes, resembling a type I interferon response. This process is associated with neuroinflammation, neuronal dysfunction, widespread RNA decay, apoptosis, and subsequent neurodegeneration [23,24,29]. The most characteristic clinical presentation typically includes a childhood-onset phenotype with progressive cerebellar ataxia, sensory axonal polyneuropathy, dysarthria, extensor plantar responses, scoliosis, pes cavus, facial myokymia and tics, gaze-evoked nystagmus, strabismus, reduced visual acuity, variable gastroparesis, intellectual disability, urinary incontinence, dystonia, and variable sensorineural hearing loss [24,30]. Sensory neuropathy may be a clue in the diagnostic evaluation of suspected SCA25, although this does not represent a pathognomonic feature [31]. Moreover, some cases have been identified with a very late onset of symptoms, featuring mildly affected individuals with mild ataxia and late-onset hearing loss. In some late-onset cases, sensory neuronopathy with absent ankle tendon reflexes may be a prominent clinical feature. Although previously classified in the clinical context of SCA under the designation SCA25, heterozygous pathogenic variants in the *PNPT1* gene may result in a very early-onset neurological presentation of spastic ataxia with significant neuro-ophthalmological involvement, as described in our case report. To date, there is no well-defined clinical–genetic correlation. It is worth noting that mitochondrial dysfunction has also been identified in other forms of Spinocerebellar Ataxia and spastic ataxias, including Friedreich’s ataxia (*FXN*), SCA type 28 (*AFG3L2*), and Autosomal Recessive Spastic Ataxia type 4 (*MTPAP*) [28,32]. Among the causes of childhood-onset HSP, it was demonstrated that among 16 patients at the Neuromuscular Clinics and Children’s and Emery Healthcare in Atlanta, US, one case (6.7%) was associated with a *PNPT1* pathogenic variant [6]. However, the inclusion of the *PNPT1* gene in most NGS genetic panels related to SCA or pure and complicated HSP is not yet routine practice, even in leading neurogenetic reference centers. 

Autosomal recessive presentations have been primarily associated with Combined Oxidative Phosphorylation Deficiency Type 13 (MIM #614932) [33] and Autosomal Recessive Deafness Type 70 with or without adult-onset neurodegeneration (MIM #614934). Autosomal recessive nonsyndromic hearing impairment has been linked to compound heterozygous and homozygous variants in the *PNPT1* gene [34,35]. Cystic leukoencephalopathy and early-onset hypomyelinating leukodystrophies have also been reported in association with biallelic *PNPT1* variants [25,36], as well as severe multisystemic presentations of Leigh syndrome and complex neurodegenerative phenotypes, including myoclonus, choreoathetosis, cerebellar ataxia, and optic atrophy [37,38]. Furthermore, in very rare contexts, complex neurological pictures have also been described, mimicking clinical and pathophysiological features that are observed in interferonopathies, such as Aicardi–Goutières syndrome [39]. Table 1 summarizes the main characteristics that are associated with autosomal recessive and dominant (or sporadic) cases of *PNPT1*-related disorders, and it presents the main differential diagnosis in relation to the clinical spectrum. 

The primary clinical prototype that was considered as a differential diagnosis for the phenotype presented by the patient in our case report, which includes spastic ataxia, ophthalmoparesis, and optic atrophy, is represented by SPG7 (MIM #607259). Initially, SPG7 was the leading neurogenetic diagnostic hypothesis for our patient. Although the majority of SPG7 cases are associated with autosomal recessive presentations and have an onset in young adulthood or childhood, forms with an occasional autosomal dominant pattern or de novo variants, and cases with late-onset after the fifth decade of life or very early-onset in infancy or with a predominant cerebellar ataxia component, have also been widely described [4,7,8,40]. Parkinsonism and dystonia may also be observed in the context of SPG7 [34,41], and in rare cases SPG7, patients may present with long-standing childhood-onset optic atrophy and minor signs of motor involvement [42]. Other forms of early-onset Hereditary Spastic Paraplegia may present with a similar clinical profile, such as SPG35 (*FA2H*), SPG79B (*UCHL1*), SPG11, and SPG15 (usually with even more complicated phenotypes) [4]. More rarely, cases of the Cerebellar Ataxia, Areflexia, Pes Cavus, Optic Atrophy, and Sensorineural Hearing Loss (CAPOS) syndrome related to the *ATP1A3* gene have been reported in association with a similar clinical picture. Other early-onset spastic ataxia conditions, like SPAX7 and SPAX4, HSP due to inherited neurometabolic disorders, and the group of sacsinopathies, including Autosomal Recessive Spastic Ataxia of Charlevoix-Saguenay (ARSACS), also constitute important differential diagnoses, although neuro-ophthalmic involvement with optic atrophy is less characteristic [7,8,43]. In the epidemiological context of the reported patient, considering his age at the onset of symptoms and Brazilian origin, the diagnostic possibility of Spastic Paraplegia, Optic Atrophy, and Neuropathy (SPOAN) syndrome (MIM #609541) should be emphasized. This is a rare autosomal recessive form with a very early onset in infancy, related to a founder effect of a homozygous 216-bp deletion in the noncoding upstream region of the *KLC2* gene (11q13.2), in populations from restricted regions in Northeastern Brazil [44,45]. 

The case reported here should also alert clinicians to the context of similarity with SCA involving neuro-ophthalmic features, such as the pigmentary retinal degeneration seen in SCA type 7 (SCA7), the classic form of group II in the ADCA classification proposed by Harding [28]. SCA7 typically manifests in early adulthood or childhood with cerebellar ataxia, signs of pyramidal tract involvement, and a possible association with movement disorders (choreoathetosis, parkinsonism, orofacial dyskinesia). It exhibits evident genetic anticipation but generally no neuropathic involvement and only very rarely with significant cognitive impairment [46,47]. Significant spasticity is not a hallmark in SCA7 presentation [47]. It is important to note that SCA presentations that are associated with *PNPT1* variants do not involve trinucleotide CAG repeat expansion diseases. Instead, they usually result from missense variants and a lack evidence of genetic anticipation. 

Considering that it is a protein that is structurally and functionally related to the mitochondria, similar to other mitochondrial contexts, such as variants in nuclear genome genes, like *MFN2*, *OPA1*, *OPA2*, *OPA10*, and mitochondrial genomes, such as some forms of Leber Hereditary Optic Neuropathy [10,11,12,32], heterozygous forms of *PNPT1* should be strongly considered during the investigation of patients with cerebellar ataxia or spastic ataxia associated with optic atrophy. It is highly recommended to conduct comprehensive genetic testing with NGS-based panels or Whole-Exome Sequencing (WES), especially in cases without an identifiable family history and a higher association with de novo variants. It is also of note that patients with heterozygous variants in the *PNPT1* gene present with a complex neurometabolic disorder with mitochondrial dysfunction and hyperactivation of neuroinflammatory pathways, but nevertheless without a well-defined diagnostic biomarker in plasma, tissues, or the cerebrospinal fluid. 

## 4. Conclusions

It is imperative that pathogenic variants in the *PNPT1* gene be considered in clinical practice for the proper diagnostic work-up of patients with early-onset spastic ataxia or spastic paraparesis that is associated with optic atrophy, regardless of the presence of a significant neurological family history. The possibility of de novo variants and autosomal dominant inheritance should be emphasized to distinguish these cases from complicated HSP, which typically have autosomal recessive inheritance. There are no cerebrospinal fluid, plasma, or neuroimaging biomarkers that can provide more suggestive clues during the diagnostic work-up of this neurometabolic disorder. 

## Figures and Tables

**Table 1:** Summary of the main clinical and genetic aspects related to autosomal recessive and dominant forms of *PNPT1* spectrum disorders.

Pattern of Inheritance (Zygosity)	Phenotypes (#MIM)	Age at Onset/Clinical Course	Family History/*PNPT1* Variants	Spasticity/Cerebellar Ataxia	Neuromuscular Involvement/Neurophysiological Features	Sensorineural Deafness/Neuro-Ophthalmological Disturbance	Other Movement Disorders (Parkinsonism, Chorea, Dystonia, Myoclonus)	Neuropsychiatric and Cognitive Involvement/Neuroimaging Findings	Skeletal Features/Gastrointestinal and Genitourinary Involvement
**Autosomal recessive forms** (compound heterozygous or homozygous variants)	**Combined Oxidative Phosphorylation Deficiency type 13 (COXPD13) (MIM #614932)**	Infancy onset; rapidly progressive and severe, then nonprogressive after	Consanguinity; positive history in recurrence/variants inside the catalytic core and active site of the protein—affects multimerization and destabilizes the mutated protein	++/++	Myopathy, axial hypotonia, dysphagia; sensory neuropathy; axonal and demyelinating sensory neuropathy; autonomic neuropathy	++/optic atrophy, macular pigmentary changes; nystagmus, strabismus, cataracts; chorioretinal defects	Dystonia, choreoathetosis, dyskinesia; myoclonus	Neurological regression (more marked in epileptic encephalopathy forms); global developmental delay/Cerebellar atrophy, leukodystrophy (including cystic leukodystrophy and hypomyelination), thin corpus callosum; Leigh syndrome-like pattern (caudate head, putamen); optic tract atrophy; possible lactate peak on MR spectroscopy	Scoliosis/Gastroparesis; constipation; Gastroesophageal reflux
	Differential diagnosis: Other primary respiratory chain complex defects (oxidative phosphorylation defects); organic acidurias (i.e., glutaric aciduria type 1); Leigh syndrome; infantile neuroaxonal dystrophy (*PLA2G6*); Neuronal Ceroid Lipofuscinosis; Sphingolipidosis								
	**Autosomal Recessive Deafness with or without adult-onset Neurodegeneration (DFNB70) (MIM #614934)**	Congenital hearing loss, neurological adulthood; progressive	Consanguinity; positive history in recurrence/variants outside the catalytic core of the protein—hypofunctional protein and abnormal PNPase trimerization	+/++	Late-onset dysphagia; late-onset myopathy; unremarkable neurophysiological studies	+++/Late-onset optic atrophy	Dystonia	Cognitive decline; mood disorders, obsessive compulsive disorder; psychotic features/normal, mild cortical cerebral or cerebellar atrophy	---/late-onset urinary incontinence
	Differential diagnosis: Mitochondrial DNA disorders (MELAS syndrome, *MT-TL1*, *MT-ND5*); Mohr–Tranebjaerg syndrome; Usher syndrome spectrum; Riboflavin transporter defects; Wolfram and Wolfram-like syndromes; MEGDEL syndrome; Arts syndrome; Neuroacanthocytosis; Late-onset Friedreich ataxia								
**Autosomal dominant form or sporadic cases** (heterozygous variants)	**Spinocerebellar Ataxia type 25 (SCA25) (MIM #608703)**	Childhood or juvenile onset; progressive	Positive family history, despite some sporadic cases; incomplete penetrance/splicing and nonsense variants with premature stop codon	+++/+++	Sensory neuropathy; absent tendon reflexes; axonal sensory neuropathy (ganglionopathy)	+/Strabismus; ophthalmoparesis; gaze-evoked nystagmus; slow saccadic pursuit; optic atrophy; late-onset diplopia	Facial tics; facial myokymia; dystonia; late-onset head tremor	Cognitive decline; intellectual disability (minor)/normal, cerebellar atrophy	Pes cavus, scoliosis/Gastroparesis; constipation; urinary urgency or incontinence
	Differential diagnosis: *SPG7*; SPOAN syndrome; SCA7; SPG35, SPG79B, *SPG11*, SPG15; CAPOS syndrome; SPAX7, SPAX4; ARSACS; nuclear genome-associated mitocondrial disorders (*MFN2*, *OPA1*, *OPA2*, *OPA10*); mitochondrial DNA mutations								

**Legends**: +: mild; ++: moderate; +++: severe; ARSACS: Autosomal Recessive Spastic Ataxia of Charlevoix-Saguenay; CAPOS: Cerebellar Ataxia, Areflexia, Pes Cavus, Optic Atrophy, and Sensorineural Hearing Loss; MEGDEL: 3-methylglutaconic aciduria with Deafness, Encephalopathy, and Leigh-like syndrome; MELAS: Mitochondrial Myopathy, Encephalopathy, Lactic Acidosis, and Stroke-like Episodes; MR: Magnetic Resonance; PNPase: Polynucleotide Phosphorylase; SCA: Spinocerebellar Ataxia; SPAX: spastic ataxia; SPOAN: Spastic Paraplegia, Optic Atrophy, and Neuropathy.

## Data Availability

The data presented in this study are available on request from the corresponding author.

## Abbreviations

ACMG	American College of Medical Genetics and Genomics
ADCA	Autosomal Dominant Cerebellar Ataxias
AMP	Association for Molecular Pathology
ARSACS	Autosomal Recessive Spastic Ataxia of Charlevoix-Saguenay
CAPOS	Cerebellar Ataxia, Areflexia, Pes Cavus, Optic Atrophy, and Sensorineural Hearing Loss
HSP	Hereditary Spastic Paraplegia
ICARS	International Cooperative Ataxia Rating Scale
IEM	Inborn Errors of Metabolism
MRI	Magnetic Resonance Imaging
mtRNA	Mitochondrial RNA
NGS	Next-Generation Sequencing
PNPase	Polynucleotide phosphorylase
SARA	Scale for the Assessment and Rating of Ataxia
SCA	Spinocerebellar Ataxia
SCA7	Spinocerebellar Ataxia type 7
SCA25	Spinocerebellar Ataxia type 25
SPAX	Spastic ataxias
SPG	Spastic Paraplegia
SPOAN	Spastic Paraplegia, Optic Atrophy, and Neuropathy
SPRS	Spastic Paraplegia Rating Scale
SWI	Susceptibility weighted imaging
WES	Whole-Exome Sequencing
